# Percolation, sliding, localization and relaxation in topologically closed circuits

**DOI:** 10.1038/srep22735

**Published:** 2016-03-10

**Authors:** Daniel Hurowitz, Doron Cohen

**Affiliations:** 1 Department of Physics, Ben-Gurion University of the Negev, Beer-Sheva, Israel

## Abstract

Considering *a random walk in a random environment* in a topologically closed circuit, we explore the implications of the percolation and sliding transitions for its relaxation modes. A complementary question regarding the “delocalization” of eigenstates of non-hermitian Hamiltonians has been addressed by Hatano, Nelson, and followers. But we show that for a conservative stochastic process the implied spectral properties are dramatically different. In particular we determine the threshold for under-damped relaxation, and observe “complexity saturation” as the bias is increased.

The original version of Einstein’s Brownian motion problem is essentially equivalent to the analysis of a simple *random walk*. The more complicated version of *a random walk on a disordered lattice*, features a percolation-related crossover to variable-range-hopping, or to sub-diffusion in one-dimension^1^. In fact it is formally like a resistor-network problem, and has diverse applications, e.g. in the context of “glassy” electron dynamics^2^,^3^. But more generally one has to consider Sinai’s spreading problem^4^,^5^,^6^,^7^, aka *a random walk in a random environment*, where the transition rates are allowed to be asymmetric. It turns out that for any small amount of disorder an unbiased spreading in one-dimension becomes sub-diffusive, while for bias that exceeds a finite threshold there is a *sliding transition*, leading to a non-zero drift velocity. The latter has relevance e.g. for studies in a biophysical context: population biology^8^,^9^, pulling pinned polymers and DNA denaturation^10^,^11^ and processive molecular motors^12^,^13^.

The dynamics in all the above variations of the random-walk problem can be regarded as a stochastic process in which a particle hops from site to site. The rate equation for the site occupation probabilities ***p*** = {*p*_*n*_} can be written in matrix notation as


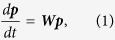


involving a matrix ***W*** whose off-diagonal elements are the transition rates *w*_*nm*_, and with diagonal elements −*γ*_*n*_ such that each column sums to zero. Assuming near-neighbor hopping the ***W*** matrix takes the form


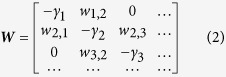


In Einstein’s theory ***W*** is symmetric, and all the non-zero rates are the same. Contrary to that, in the “glassy” resistor-network problem (see Methods) the rates have some distribution *P*(*w*) whose small *w* asymptotics is characterized by an exponent *α*, namely *P*(*w*) ∝ *w*^*α*−1^ for small *w*. To be specific we consider


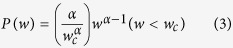


The conductivity of the network *w*_∞_ is sensitive to *α*. It is given by the harmonic average over the *w*_*n*_, reflecting serial addition of connectors. It comes out non-zero in the percolating regime (*α* > 1). For the above distribution *w*_∞_ = [(*α* − 1)/*α*]*w*_*c*_.

In Sinai’s spreading problem ***W*** is allowed to be asymmetric. Accordingly the rates at the *n*th bond can be written as 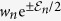 for forward and backward transitions respectively. For the purpose of presentation we assume that the stochastic field 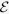 is box distributed within [*s* − *σ*, *s* + *σ*]. We refer to *s* as the bias: it is the pulling force in the case of depinning polymers and DNA denaturation; or the convective flow of bacteria relative to the nutrients in the case of population biology; or the affinity of the chemical cycle in the case of molecular motors.

Our interest is in the relaxation dynamics of finite *N*-site ring-shaped circuits^14^,^15^, that are described by the stochastic equation equation (1). The ring is characterized by its so-called affinity,


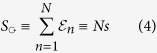


The *N* sites might be physical locations in some lattice structure, or can represent steps of some chemical-cycle. For example, in the Brownian motor context *N* is the number of chemical-reactions required to advance the motor one pace. We are inspired by the study of of non-Hermitian quantum Hamiltonians with regard to vortex depinning in type II superconductors^16^,^17^,^18^; molecular motors with *finite* processivity^19^,^20^; and related works^21^,^22^,^23^. In the first example the bias is the applied transverse magnetic field; and *N* is the number of defects to which the magnetic vortex can pin. In both examples conservation of probability is violated.

## Scope

**Figure 1:**
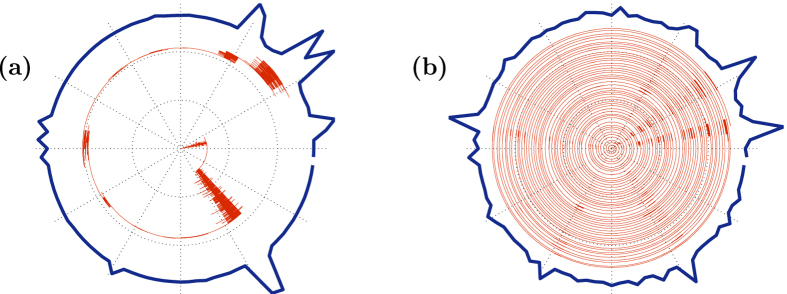
Simulated trajectory of a particle on a disordered-ring. The number of sites is *N* = 100 and the disorder strength is *σ* = 5. The radial direction is time and the angle is the position. (**a**) For small affinity (*s* = 0.88) the dynamics is over-damped. (**b**) For large affinity (*s* = 2.97) the dynamics is under-damped. The outer thick line is the steady-state distribution (see Methods).

**Figure 2:**
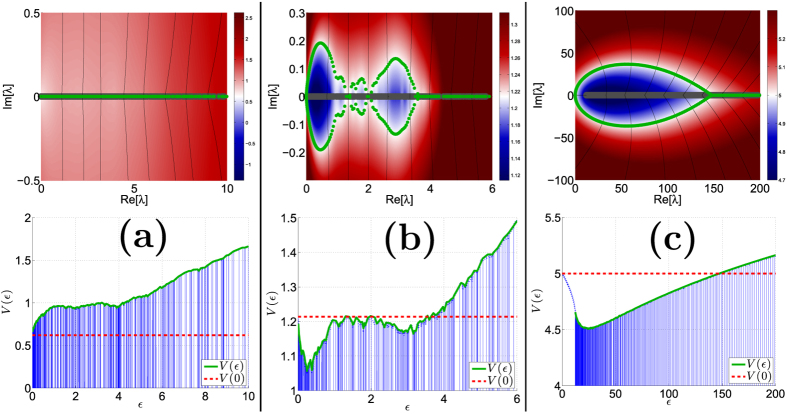
The emergence of complexity in the relaxation spectrum. The upper plot in each panel displays a representative example for a relaxation spectrum. The {*λ*_*k*_} are indicated by green points in the complex plane. The ring has *N* = 500 sites. The disorder is *σ* = 5, hence the calculated threshold values are *s*_1/2_ = 1.77 and *s*_1_ = 2.7 and *s*_∞_ = 5. In (**a**) the affinity is *s* = 1.24 < *s*_1/2_ and the spectrum is real. In (**b**) the affinity is *s* = 2.43 and the spectrum has several complex bubbles separated by real segments. In (**c**) the affinity is *s* = 10 > *s*_∞_ and the real spectrum has a gap, while the complex spectrum is a fully developed complex bubble, tangent to the origin (no gap). The “electrostatic field” that is associated with the characteristic equation is represented by a few field-lines, while the background color provides visualization of the corresponding electrostatic potential. The spectrum is obtained by looking for the intersections of the field lines with the equipotential line *V*(*z*) = *V*(0) that goes through the origin (indicated in white). The lower panels plot the potential *V*(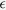) along the real axis. The horizontal dashed line is *V*(0).

**Figure 3:**
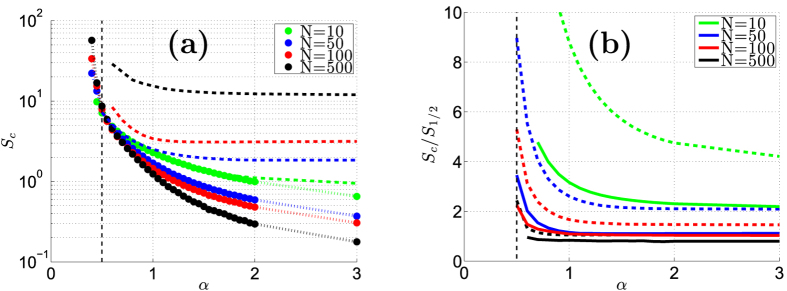
The complexity threshold. We plot *S*_*c*_ versus *α* for rings with *N* = 10, 50, 100, 500 sites, and representative values of stochastic field disorder. The dotted, dashed and solid lines are for *σ* = 0, 0.5, 1.0 respectively. Average has been taken over ~100 realizations for each data point. In panel (**a**) the data points of the *σ* = 0 curve are indicated by circles. We clearly see that the effective percolation threshold, beyond which *S*_*c*_ becomes finite, is *α* = 1/2 rather than *α* = 1. For a percolating disorder *S*_*c*_ diminishes as *N* is increased, while for marginal percolation 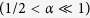 the threshold becomes *N* independent as for a clean-ring that has a single weak-link. The dashed lines in panel (**a**) are for *σ* = 0.5. In order to demonstrate that they agree with *S*_*c*_ = *Ns*_1/2_ we plot additional curves for *σ* = 1.0 in panel (**b**), and scale the vertical axis appropriately. Comparing the two panels we see that the *s*_1/2_ based prediction becomes valid once it exceeds the resistor-network threshold.

**Figure 4:**
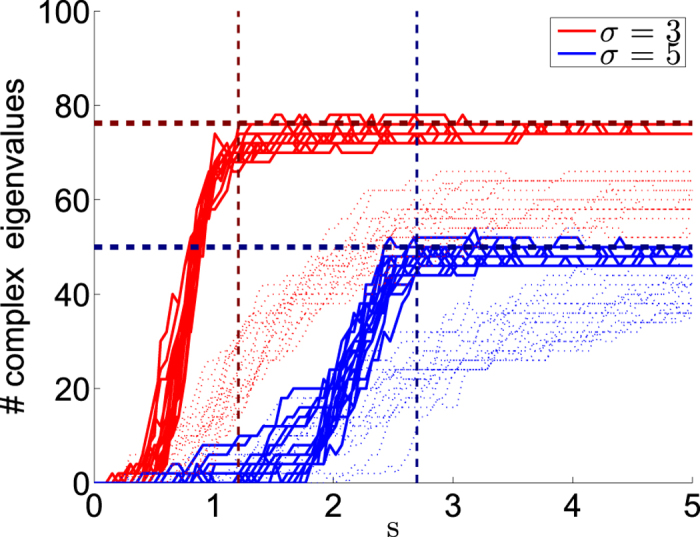
Complexity saturation. The number of complex eigenvalues is counted for a ring with *N* = 100 sites, for various values of the affinity *s*. Each red line corresponds to a different realization of field disorder with *σ* = 3 (red) and *σ* = 5 (blue). The vertical lines are the corresponding values of *s*_1_, at which the sliding transition occurs. We see that the asymptotic fraction of complex eigenvalues saturates. The horizontal dashed line are the analytical estimates of equation (15). If the lattice were continuous with Gaussian disorder, the number of complex eigenvalues would go to 100%. In the background a disordered resistor network with *α* = 0.9 is shown. The crossover is blurred and the saturation value is lower compared to equation (15).

In this article we report how the spectral properties of the matrix ***W*** depend on the parameters (*α*, *σ*, *s*), as defined above. These parameters describe respectively the resistor-network disorder, the stochastic-field disorder, and the average bias field. The eigenvalues {−*λ*_*k*_} of ***W*** are associated with the relaxation modes of the system. Due to conservation of probability *λ*_0_ = 0, while all the other eigenvalues {*λ*_*k*_} have positive real part, and may have an imaginary part as well. Complex eigenvalues imply that the relaxation is not over-damped: one would be able to observe an oscillating density during relaxation, as demonstrated in Fig. 1. The panels of Fig. 2 provide some representative spectra. As the bias *s* is increased a complex bubble appears at the bottom of the band, implying delocalization of the eigenstates. Our results for the complexity threshold *S*_*c*_ are summarized in Table 1, and demonstrated in Fig. 3. The number of complex eigenvalues grows as a function of the bias, as demonstrated in Fig. 4, but asymptotically only a finite fraction of the spectrum becomes complex. Our objective below is to explain analytically the peculiarities of this delocalization transition, to explain how it is affected by the percolation and by the sliding thresholds, and to analyze the complexity-saturation effect.

### Note about semantics

What we called above a “percolation-like transition” at *α* = 1 means that for an infinite chain, in the statistical sense, the conductivity (*w*_∞_) is zero for *α* < 1 and becomes non-zero for *α* > 1. Clearly, if the bond distribution *P*(*w*) were bi-modal (if the *w*_*n*_ were zeros or ones), we would not have in one-dimension a percolation transition^24^.

## Stochastic spreading

We first consider an opened ring, namely a disordered chain. The asymmetry can be gauged away, and ***W*** becomes similar to a symmetric matrix ***H*** (see Methods). The statistics of the off-diagonal elements of ***H*** is characterized by *α*, while the statistics of the diagonal elements is affected by *σ* and *s* too. The eigenvalues {−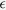
_*k*_} of ***H*** are real. In the absence of disorder they form a band [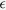
_*s*_, 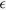
_∞_] where 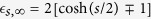. If the stochastic-field disorder has a Gaussian statistics the gap [0, 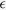
_*s*_] is closed^6^. In this case there is an analytical expression for the spectral density in terms of Bessel functions. The expression features


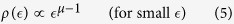


with no gap. The exponent is related to the bias via *s* = (1/2)*σ*^2^*μ*. In the present work we assume the more physically appealing log-box disorder for which the relation between *s* and *μ* is as follows (see Methods):


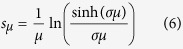


Unlike Gaussian disorder the range of possible rates is bounded, and we see that *s*_∞_ = *σ* is finite rather than infinite. For *s* > *s*_∞_ a gap opens up, meaning that 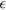
_*s*_ acquires a finite non-zero value.

In order to have a non-zero drift velocity along an *infinite* chain two conditions have to be satisfied: First of all the system has to be percolating (*α* > 1) such that its conductivity *w*_∞_ is non-zero; Additionally one requires the bias *s* to exceed the threshold *s*_1_, such that *μ* > 1. This is known as the “sliding transition”. One obtains


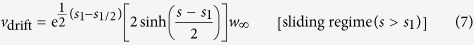


Contrary to that, in the regime *s* < *s*_1_ there is a build-up of an activation-barrier that diverges in the *N* → ∞ limit, hence the drift velocity vanishes. The above mentioned spectral properties imply that for *μ* < 1 the spreading of a distribution along an infinite chain becomes anomalously slow and goes like *x* ~ *t*^*μ*^. Concerning the second moment: for *μ* < 1/2 the diffusion coefficient is zero, reflecting sub-diffusive spreading. In the absence of bias (*μ* → 0) the spreading becomes logarithmically slow.

**Figure 5:**
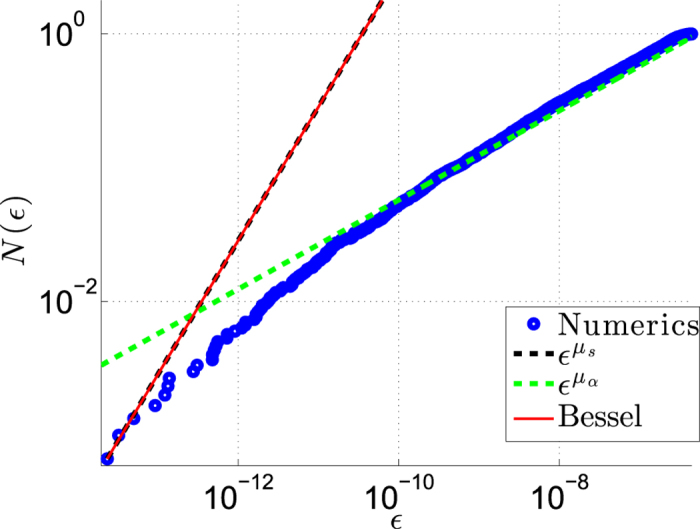
The spectrum of the associated hermitian matrix. We calculate numerically the integrated density, which counts the eigenvalues {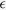
_*k*_ < 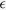} of ***H*** for a ring with *N* = 3000 sites. The system is characterized by a percolation exponent *μ* = *μ*_*α*_ = 1/3, and by a scaled affinity *μ* = *μ*_*s*_ = 1. The stochastic-field distribution is with *σ* = 2. The blue points are results of numerical diagonalization. There is a crossover from density that corresponds to *μ*_*s*_ (dashed black line), to density that corresponds to *μ*_*α*_ (dashed green line). The red line is the Bessel expression of^6^.

The absence of resistor-network-disorder formally corresponds to *α* = ∞ in equation (3), meaning that all the *w*_*n*_ have the same value. The introduction of resistor-network-disorder (*α* < ∞) modifies the spectral density equation (5) at higher energies (see Fig. 5 of the Methods for illustration). In the absence of bias, for *α* > 1, the continuum-limit approximation features *μ*_*α*_ = 1/2. This reflects a normal diffusive behavior as in Einstein’s theory of Brownian motion. Below the percolation threshold, namely for *α* < 1, normal diffusion is suppressed^1^, and the spectral exponent becomes *μ*_*α*_ = *α*/(1 + *α*) < 1/2. In the other extreme of very large bias, the diagonal disorder in ***H*** dominates, leading to trivially localized eigenstates. Hence for very large bias we simply have *μ* = *α* irrespective of the percolation aspect.

The conclusion of this section requires a conjecture that is supported by our numerical experience (we are not aware of a rigorous derivation): As the bias *s* is increased, the exponent *μ* becomes larger, as implied by equation (6), but it cannot become larger than *α*. We shall use this conjecture in order to explain the observed implications of resistor-network-disorder.

## Relaxation

We close an *N*-site chain into a ring and wonder what are the relaxation modes of the system. The starting point of our analysis is the characteristic equation for the eigenvalues of ***W***. Assuming that we already know what are the eigenvalues of the associated symmetric matrix ***H***, the characteristic equation takes the form^25^ (see Methods)


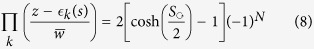


where 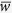 is the geometric average of all the rates. The bias *s* affects both the 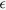
_*k*_ and the right hand side. This equation has been analyzed in^18^ in the case of a non-conservative matrix ***W*** whose diagonal elements *γ*_*n*_ are *fixed*, hence the 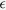
**_*k*_(*s*) there do not depend on *s*. Consequently, as *s* of equation (4) is increased beyond a threshold value *s*_*c*_, the eigenvalues in the middle of the spectrum become complex. As *s* is further increased beyond some higher threshold value, the entire spectrum becomes complex. As already stated in the introduction, this is not the scenario that is observed for our conservative model. Furthermore we want to clarify how the percolation and sliding thresholds are reflected.

Already at this stage one should be aware of the immediate implications of conservativity. First of all *z* = *λ*_0_ = 0 should be a root of the characteristic equation. The associated eigenstate is the non-equilibrium steady state (NESS), which is an extended state (see Methods). In fact it follows that the localization length has to diverge as *λ* → 0. This is in essence the difference between the conventional Anderson model (Lifshitz tails at the band floor) and the Debye model (phonons at the band floor). It is the latter picture that applies in the case of a conservative model.

## Electrostatic picture

In order to gain insight into the characteristic equation we define an “electrostatic” potential by taking the log of the left hand side of equation (8). Namely,


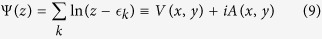


where *z* = *x* + *iy*, and for simplicity of presentation we set here and below the units of time such that 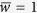. The constant *V*(*x*, *y*) curves correspond to potential contours, and the constant *A*(*x*, *y*) curves corresponds to stream lines. The derivative Ψ′(*z*) corresponds to the field, which can be regarded as either electric or magnetic field up to a 90 deg rotation. Using this language, the characteristic equation equation (8) takes the form


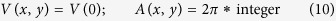


Namely the roots are the intersection of the field lines with the potential contour that goes through the origin (Fig. 2). We want to find what are the conditions for getting a real spectrum from equation (10), and in particular what is the threshold *s*_*c*_ for getting complex eigenvalues at the bottom of the spectrum. We first look on the potential along the real axis:


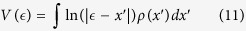


In regions where the {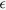
_*k*_} form a quasi-continuum, one can identify (1/*N*)*V*(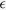) as the Thouless expression for the inverse localization length^18^. The explicit value of *V*(0) is implied by equation (8), namely 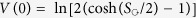. For a charge-density that is given by equation (5), with some cutoff 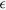
_*c*_, the derivative of the electrostatic potential at the origin is (see Methods)


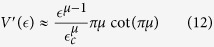


One observes that the sign of *V*′(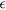) is positive for *μ* < 1/2, and negative for *μ* > 1/2. Some examples are illustrated in Fig. 2. Clearly, if the envelope of *V*(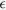) is above the *V* = *V*(0) line, then the spectrum is real, and the *λ*_*k*_ are roughly the same as the 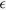
_*k*_, shifted a bit to the left.

From the above it follows that the threshold *s*_*c*_ for the appearance of a complex quasi-continuum is either *V*(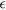
_*s*_) < *V*(0) or *V*′(0) < 0, depending on whether *ρ*(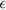) is gapped or not. In the latter case it follows from equation (12) that *s*_*c*_ = *s*_1/2_. We note that for the Gaussian model of^6^ one obtains *V*(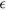 → ∞) = const, implying that the entire spectrum would go from real to complex at *s* = *s*_1/2_. In general this is not the case: the complex spectrum typically forms a “bubble” tangent to the origin, or possibly one may find some additional bubbles as in Fig. 2b (upper plot).

## Resistor-network disorder

The prediction *S*_*c*_ = *Ns*_1/2_ assumes full stochastic-field disorder over the whole ring. One may have the impression that this result suggests *S*_*c*_ = 0 in the absence of stochastic field disorder, because *s*_1/2_ = 0 for *σ* = 0. We shall argue below that this is a false statement. Clearly the prediction *S*_*c*_ = *Ns*_1/2_ is irrelevant if one link is disconnected (say *w*_1_ = 0). In the latter case one would expect *S*_*c*_ = ∞. Naively an infinite *S*_*c*_ might be expected throughout the non-percolating regime (*α* < 1). But we shall argue that this is a false statement too.

Consider first a clean ring. Recall that it features a continuous spectral density *ρ*(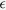) that is supported by [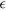
_*s*_, 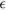
_∞_]. An isolated defected bond contributes an isolated eigenvalue outside of the band. This is like having an impurity. A detailed example for this, is presented in the Methods section, where we establish that for a weak-link *S*_*c*_ is finite, and independent of *N*. Similar analysis can be carried-out for other types of isolated defects.

Full resistor-network disorder (*α* < ∞ with *σ* = 0) can be regarded as having some distribution of “weak-links” along the ring. We can speculate that for large *N* there are two limits: either *S*_*c*_ → ∞ or *S*_*c*_ → 0 depending on whether the ring is percolating or not. Our numerical results are presented in Fig. 3a. Surprisingly the effective percolation threshold is not *α* = 1, but *α* = 1/2. The threshold *S*_*c*_ becomes infinite only if *α* < 1/2. We are able to predict this numerical observation using the electrostatic picture: In the regime *α* < 1/2 the spectral density *ρ*(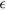) is characterized by a an exponent *μ* < 1/2. Namely it goes from *μ* = *α*/(1 + *α*) < 1/2 for small 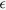, to *μ* = *α* < 1/2 for large 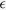. Consequently *V*(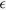) becomes a monotonic increasing function, and it follows from the the reasoning of the previous section that all eigenvalues are real.

For a percolating but disordered resistor-network (1 < *α* < ∞ but *σ* = 0) we expect *S*_*c*_ ∝ 1/*N*^1/2^, see Methods. The marginally-percolating regime (1/2 < *α* < 1) is conceptually like having sparsely distributed weak-links. Accordingly, as *α* → 1/2 the threshold *S*_*c*_ becomes independent of *N*. These predictions are confirmed numerically in Fig. 3a. The additional numerical results that are presented in Fig. 3b demonstrate what happens if we add stochastic field disorder: The prediction *S*_*c*_ = *Ns*_1/2_ becomes valid once it exceeds the resistor-network threshold.

## Complexity saturation

The characteristic equation for the eigenvalues is given by equation (8). In the nonconservative case, the eigenvalues of ***H*** do not depend on *s*, thus raising *s* will eventually make the entire spectrum complex. For a conservative matrix, however, *V*(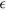) is also a function of *s*, so increasing *s* raises *V*(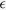) at the same rate. Taking *s* to be as large as desired, the eigenvalues of *H* become trivially 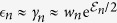, and the equation *V*(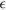) = *V*(0) for the upper cutoff 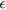
_*c*_ of the complex energies takes the form


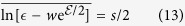


It is natural to write the stochastic field as 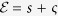, such that *ς*∈[−*σ*, +*σ*]. For the purpose of presentation we assume that *w* = 1. Then the spectrum stretches from 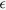
_*s*_ = e^(*s*−*σ*)/2^ to 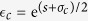, where *σ*_*c*_ is the solution of


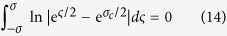


It follows that the fraction of complex eigenvalues is


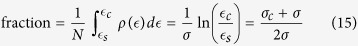


We demonstrate the agreement with this formula in Fig. 4. We plot there also what happens if resistor-network disorder is introduced. We see that for small *α* the crossover is not as sharp and the saturation value is lower than equation (15) as expected from equation (13).

## Discussion

We have shown that the relaxation properties of a closed circuit (or chemical-cycle), whose dynamics is generated by a conservative rate-equation, is dramatically different from that of a biased non-hermitian Hamiltonian. The transition to complexity (under-damped dynamics, see Fig. 1b) depends on the type of disorder as summarized in Table 1. Surprisingly it happens at *α* = 1/2 before the *α* = 1 percolation transition, and at *μ* = 1/2 before the *μ* = 1 sliding transition. Further increasing the bias does not lead to full delocalization, instead a “complexity saturation” is observed.

In our analysis, we were able to bridge between the works of Hatano, Nelson, Shnerb, and followers, regarding the spectrum of non-hermitian Hamiltonians; the works of Sinai, Derrida, and followers, regarding random walks in random environments; and the works of Alexander and co-workers regarding the percolation related transition in “glassy” resistor network systems. Furthermore we have uncovered a related misconception concerning processive molecular motors, contradicting a widespread conjecture regarding the equivalence to a uni-directional hopping model with a broad distribution of dwell times (see below).

Spreading processes in disordered systems have been widely studied. In the pioneering work of Derrida^5^, the velocity and diffusion coefficient in the steady state were found by solving the rate equation for an *N*-site periodic lattice, and then taking the limit *N* → ∞. In^6^,^26^ the same results have been obtained using a Green function method, which requires averaging over realizations of disorder rather than considering a periodic chain. The main shortcoming of both approaches is that going beyond the steady-state is very difficult. Yet another approach, followed by Kafri, Lubensky and Nelson^20^, is to utilize the perspective of Hatano, Nelson and Shnerb^16^,^17^,^18^, who studied the entire spectrum by diagonalization of the pertinent non-hermitian matrix. This method is especially appealing if one is interested in the behavior of the system at long times. Additionally, this method accounts for a closed topology, an aspect disregarded in the other methods.

In this context we would like to highlight the study of the long-time behavior of processive molecular motors, such as RNAp or DNAp, moving along heterogeneous DNA tracks^20^. The so-called *stall force* of the motor corresponds to a bias given by *s*_1_. For *s* < *s*_1_ the drift becomes anomalous. The common wisdom was that the relaxation spectrum remains complex for any *s*, but with an anomalous density for *s* < *s*_1_. This statement had been supported by a conjectured equivalence to a uni-directional hopping model with a broad distribution of dwell times, that has been proposed by Bouchaud, Comtet, Georges, and Le Doussal^6^. Thus it has been concluded that *reality* requires finite-processivity. In the present work we have established that the reality of the spectrum (over-damped relaxation) prevails also for infinite-processivity, but the threshold is *s*_1/2_ rather than *s*_1_. Furthermore we have provided insights regrading various ingredients that affect the breakdown of reality; the emergence of complexity; and its ultimate saturation.

## Methods

### The percolation threshold

An example where the percolation issue arises is provided by the analysis of relaxation in “glassy” networks^2^,^3^, where the sites are distributed randomly in space, and the rates depend exponentially on the inter-site distance, namely *w* ∝ exp(−*r*/*ξ*). In such type of model there is a percolation-related crossover to variable-range-hopping^27^. But in one-dimension there is a more dramatic crossover to sub-diffusion^1^. The statistics of the inter-site distances is Poisson Prob(*r*) ∝ exp(−*r*/*a*), where *a* is the mean spacing, and therefore *α* = *ξ*/*a* in equation (6). The diffusion coefficient is the harmonic average over *w*_*n*_, reflecting serial addition of connectors. It becomes zero for *α* < 1.

The percolation control-parameter *α* is reflected in the exponent *μ* that characterizes the spectral function equation (5). As explained in the main text, the exponent *μ* is further affected by the bias *s*. See Fig. 5 for illustration.

### The NESS formula

Following the derivation in^14^ the explicit formula for the NESS is


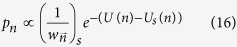


where *U*(*n*) is the stochastic potential that is associated with the stochastic field such that 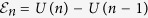. The transitions in the drift-wise direction are 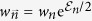, and the subscript *s* indicates drift-wise smoothing over a length scale 1/*s*. In the absence of bias the smoothed functions are constant and we get the canonical equilibrium state.

### Handling ***W***

Define the diagonal matrix ***U*** = diag{*U*(*n*)}. The stochastic field can be made uniform, as in^18^, by performing a similarity transformation 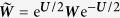, leading to


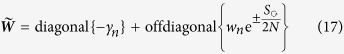


where the “±” are for the forward and backward transitions respectively. Note that the *s*-dependent statistics of the 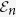 is still hiding in the diagonal elements. The associated symmetric matrix ***H*** is defined by setting 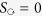. Then one can define an associated spectrum {−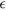
_*k*_}. For an open chain setting 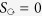 can be regarded as a gauge transformation of an imaginary vector potential. For a closed ring 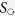 is like an imaginary Aharonov-Bohm flux, and cannot be gauged away. For the spectral determinant the following expression is available^25^:


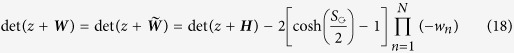


This leads to the characteristic equation, equation (8).

### Finding *s*_*μ*_

The cummulant generating function of the stochastic field can be written as *g*(*μ*) = (*s* − *s*_*μ*_)*μ*, where the *s*_*μ*_ are defined via the following expression:


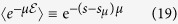


If the stochastic field has normal distribution with standard deviation *σ*, then *s*_*μ*_ = (1/2)*σ*^2^*μ*. For our log-box distribution equation (6) applies. The finite value of *s*_∞_ reflects that 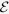 is bounded.

### Finding *V*′(0)

To derive equation (12) we assume an integrated density of states that corresponds to equation (5), namely, 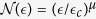, where 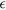
_*c*_ is some cutoff that reflects the discreteness of the lattice. After integration by parts the electrostatic potential along the real axis is given by


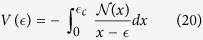


While calculating the derivative we assume 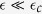, hence taking the upper limit of the scaled integral as infinity:


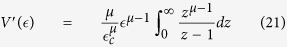



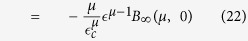


where *B*_*u*_(*a*, *b*) is the Incomplete Euler Beta function. Taking the Cauchy principal part we get


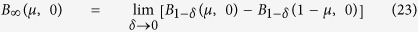



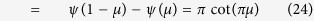


where *ψ*(*z*) is the digamma function, and the last equality has been obtained by the reflection formula.

### Finding *S*_*c*_ due to a weak-link

**Figure 6:**
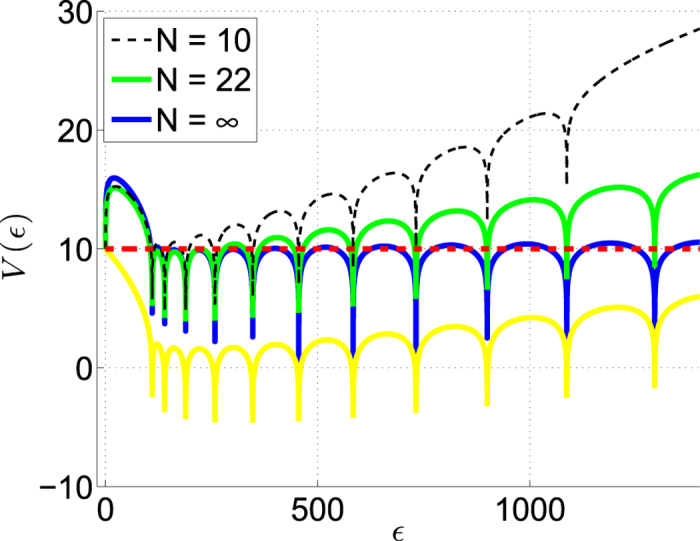
Graphical illustration of the the characteristic equation for a ring with a weak link. The red line is *V*(0). The blue line is *V*(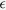) as deduced from the LHS of equation (25) with *L* = 1, and *g* = 10^−3^ and 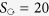. The yellow line is an attempted reconstruction of *V*(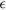) from the first *N* = 22 roots 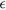
_*k*_ of the 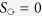 equation. The green line is a proper reconstruction that takes into account an impurity term 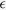
_0_. The deviation from the blue line for large *k* is due to finite truncation: compare the *N* = 10 line with the *N* = 22 line.

We consider a clean ring of length *L* = *Na* with lattice spacing *a* and identical bonds (*w*_*n*_ = 1). We change one bond into a weak link 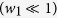. This setup can be treated exactly in the continuum limit, where equation (1) corresponds to a diffusion equation with coefficient *D*_0_ = *wa*^2^ and drift velocity *v*_0_ = *sD*_0_. The weak link corresponds to a segment where the diffusion coefficient is 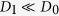. Using transfer matrix methods we find the characteristic equation


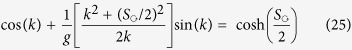


where 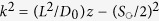, and *g* = (*D*_1_/*D*_0_)/(*a*/*L*). We have taken here the limit *a* → 0, keeping 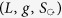 constant. The equation is graphically illustrated in Fig. 6. All the roots are real solutions provided the envelope of the left-hand-side (LHS) lays above the right-hand-side (RHS). The minimum of the envelope of the LHS is obtained at 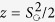. Consequently we find that the threshold *S*_*c*_ obeys


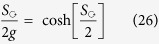


provided 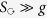, which is self-justified for small *g*. The solution is given in terms of the Lambert function, namely 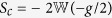, which determines 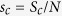.

The characteristic, equation equation (25), parallels the discrete version equation (8), with a small twist that we would like to point out. Naively one would like to identify ln[2(LHS − 1)], up to a constant, with 
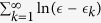
, where the 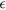
**_*k*_ are the roots of equation (25) with 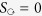 in the RHS. This is tested in Fig. 6, and we see that there is a problem. Then one realizes that in fact an additional *k* = 0 term with 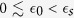 is missing. Going back to the discrete version it corresponds to an impurity-level that is associated with a mode which is located at the weak-link. While taking the limit *a* → 0 this level becomes excluded. Adding it back we we see that the agreement between equation (8) and equation (25) is restored. The residual systematic error as *k* becomes larger is due to finite truncation of the number of roots used in the reconstruction. Making the approximation ln(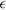
_*s*_ − 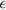
_0_) ≈ ln[(*s*/2)^2^], and noting that *g* ∝ *N*, it is verified that the equation *V*(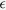
_*s*_) = *V*(0) for the complexity threshold is consistent with equation (26).

### Finding *S*_*c*_ for resistor-network disorder

In the absence of stochastic field disorder, considering a percolating ring with *α* > 1, the threshold for complexity cannot be determined by the condition *V*′(0) < 0 with equation (12), because for *μ*_*α*_ = 1/2 we get formally *V*′(0) = 0. Rather the threshold for complexity is determined by the condition *V*(0+) < *V*(0), where *V*(0+) is the the value of of *V*(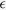
**) in the vicinity of 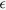 ~ 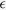
**_1_. Recall that by the Thouless expression (1/*N*)*V*(*ϵ*) has been identified as the inverse localization length^18^. We are dealing here with a “conservative matrix” where the localization diverges at the potential floor as in the Debye model. It is well known that in the Debye model (1/*N*)*V*(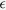) ∝ *ω*^2^ where 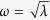 corresponds to the frequency of the phonons. Setting *ω*_1_ ∝ 1/*N* and *V*(0) ≈ (*S*/2)^2^ we conclude that *S*_*c*_ ∝ 1/*N*^1/2^.

## Additional Information

**How to cite this article**: Hurowitz, D. and Cohen, D. Percolation, sliding, localization and relaxation in topologically closed circuits. *Sci. Rep.* **6**, 22735; doi: 10.1038/srep22735 (2016).

## Figures and Tables

**Table 1:** The complexity threshold for different types of disorder (aka delocalization transition).

Type of disorder	Parameters		*S*_*c*_ for large *N*	Remarks
Resistor-network	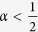	*σ* = 0	*S*_*c*_ = ∞	non-percolating (“disconnected ring”)
Resistor-network	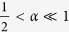	*σ* = 0	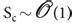	residual percolation (“weak link”)
Resistor-network	*α* > 1	*σ* = 0	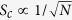	percolating (conductivity *w*_∞_ > 0)
Stochastic field	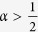	*σ* > 0	*S*_*c*_ ≈ *Ns*_1/2_	lower than sliding threshold at *Ns*_1_

We distinguish between resistor network disorder (*α* < ∞) and stochastic field disorder (*σ* > 0). The threshold *s*_1/2_ is determined by the the latter. It is smaller than the *s*_1_ threshold of the sliding transition. Note that the thresholds *s*_*μ*_ depend neither on *N* nor on *α*.
